# ‘Opening’ the closed method: military-civilian exchanges and transatlantic circulations in Manuel Bastos’ early work on compound fractures (1909–24)

**DOI:** 10.1017/mdh.2026.10060

**Published:** 2026-07

**Authors:** Francisco Javier Martinez, Carlos Murillo-Arribas

**Affiliations:** 1 University of Zaragoza, Faculty of Medicine, Zaragoza, Spain; 2 Hospital Clínico Universitario Lozano Blesa, Zaragoza, Spain

**Keywords:** Closed method, Hispano-Americanism, Manuel Bastos, Pedro Chutró, Rif War, Traumatology

## Abstract

The closed method for the treatment of compound fractures of the limbs emerged and popularised during the interwar period. The historiography on this procedure sustains an essentially Anglo-Saxon narrative focusing on contributions by the American surgeon Winnett H. Orr during the First World War and the Spanish Josep Trueta during the Spanish Civil War and his exile in Britain. This paper aims to ‘open’ this story by reconstructing the early work of another Spanish surgeon: Manuel Bastos. Although originally an army medical officer, Bastos specialised in the treatment of limb fractures in a dual military-civilian context. On the one hand, during successive assignments to the Spanish Protectorate in Morocco, he familiarised with the management of gunshot wounds. On the other hand, he specialised in the treatment of tuberculosis humerus fractures in children at the *Instituto Rubio* in Madrid. The visit to Spain of the Argentinian surgeon Pedro Chutró, who had acquired a great prestige in First World War Paris for his approach to fractures and osteomyelitis, and the escalation of the Moroccan campaigns to the so-called Rif War (1921–27) gave Bastos the opportunity, the idea, and the courage to develop a closed treatment of humerus fractures in soldiers. Chutró’s influence on Bastos persisted in the context of the Hispano-Americanist policy embraced in mid-twentieth-century Spain. Ultimately, this study questions the understanding of the closed method as a single, univocally traceable procedure, suggesting instead parallel versions emerging in different sites and transforming themselves and influencing each other as they circulated globally.

## Introduction

In international medical historiography, the authorship of the closed method, a surgical procedure for the treatment of compound fractures of the limbs that became widely accepted and practiced during the interwar period, has been essentially attributed to the surgeons Hiram Winnet Orr (West Newton, PA, 1877–Rochester, MN, 1956) and Josep Trueta Raspall (Barcelona, 1897–1977). Orr was the pioneer who, in the early 1920s, after his experience with the American Expeditionary Force in France during the First World War (1914–18), established a method consisting of a precise sequence of steps: Friedrich technique (opening and debridement of the wound); disinfection with Dakin-Carrel solution; filling with Vaseline gauze; immobilisation of the limb with plaster of Paris over a gauze bandage.^1^ However, many of his colleagues in the United States and Great Britain failed to endorse the new procedure. The Spanish Civil War (1936–39) would hasten its recognition, both due to its massive application and to the introduction of certain changes, especially the indication of applying it in the very first hours after the fracture had occurred. French doctors observing this treatment in hundreds of Republican wounded combattants who crossed the border in the retreat following the occupation of Barcelona by General Franco’s forces in February 1939 called it, with a certain contempt, ‘Spanish method’^2^.

The successful experiences of the Spanish Civil War would mainly get international projection through Great Britain. Exiled in this country, Josep Trueta’s work was quickly endorsed by the prestigious Oxford surgeon G.R. Girdlestone and published in journals such as *The Lancet* ^3^ and the *British Medical Journal.* ^4^ Trueta also disseminated his findings in the classic treatise *Treatment of War Wounds and Fractures: With Special Reference to the Closed Method as Used in the War in Spain*,^5^ whose American edition was prefaced by Orr himself.^6^ He set out his own five-point ‘programme’ for the treatment of open fractures: early surgical intervention; wound cleansing; wound excision; application of drainage; immobilisation with plaster.^7^ The Spanish surgeon traced the ultimate origin of this programme to his long experience in the treatment of industrial accidents dating back to the late 1920s, although the cases he presented in his publications in English were those wounded in Barcelona’s air raids and those evacuated from various war fronts to his service at the General Hospital of Catalonia. The British army’s adoption of Trueta’s programme during the Second World War led the ‘Orr method’ to being called ‘Orr-Trueta’.^8^ Since the 1970s, accounts sympathetic with or emanating from Catalan nationalism have referred to it simply as ‘Trueta’s method’ or claimed its ‘undisputable’ paternity for the Barcelona surgeon.^9^

**Figure 1.**
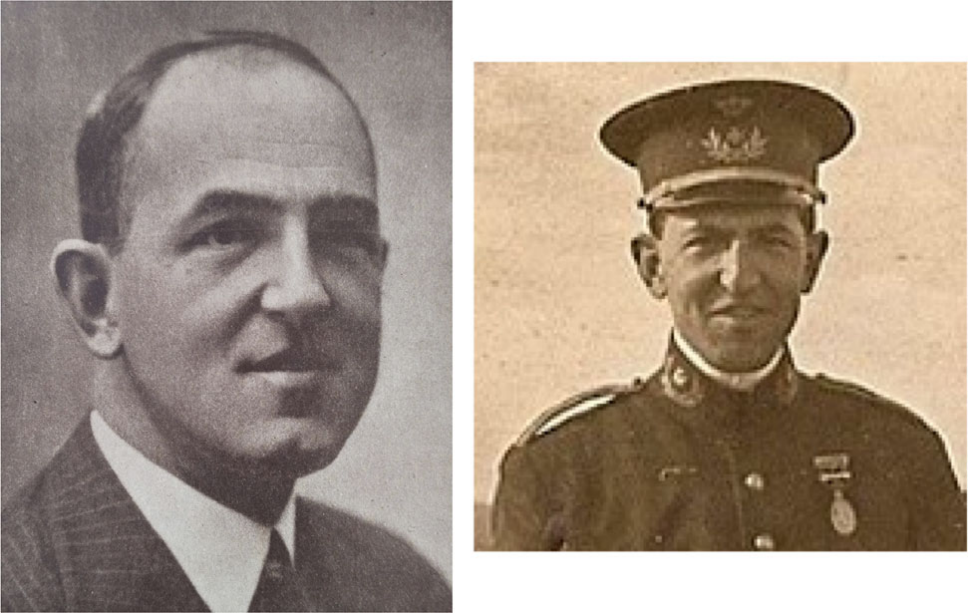
The young Manuel Bastos as civilian doctor (left) and army medical officer (right). Sources: “Manuel Bastos Ansart. Fotografies”. Galeria de Metges Catalans, https://www.galeriametges.cat/galeria-fotografies.php?icod=GDH#PrettyPhoto[gallery]/0/ [accessed, 21 August 2025]; Manuel Bastos, Los mecanismos del movimiento en el hombre y en los animales (Madrid: La Lectura, 1927). Public domain.

The central role played by Trueta and the Spanish Civil War cannot hide the fact that the story of the closed method has remained an essentially Anglo-Saxon narrative centred on the two great world conflicts of the twentieth century. The surgical traditions and the conflicts of other countries, with their corresponding actors, have been left out of a picture they could nevertheless enrich and substantially modify.^10^ Their consideration could also provide valuable insights on general questions dealt with in the history of science in recent decades, such as the relation between wars and scientific ‘progress’^11^ or the global circulation of knowledge and practices.^12^ We will engage with all these issues through a study of the Spanish army surgeon Manuel Bastos (Zaragoza 1887–Barcelona 1973) (Figure 1) and of his treatment of compound fractures in an early phase of his career, especially during his service at Morocco’s Rif War (1921–27). Bastos has received considerable attention in Spain, mostly, but not exclusively,^13^ from military medical historians and doctors, who have championed the label ‘Orr-Bastos-Trueta method’^14^ as a means to acknowledge his contributions. However, most analyses of his work have been superficial. It has been claimed without sufficient documentary evidence that Bastos already employed ‘a distant precedent’^15^ of the occlusive technique in the early campaigns in Morocco in the 1910s before properly applying it when the Rif War broke out in July 1921.^16^ The treatment of ‘a large number of open fractures in this conflict, mostly caused by bullets’^17^ would have allowed Bastos to improve ‘the closed method extraordinarily’^18^ on the basis of the principle that ‘rigorous immobilisation is a fundamental requisite […] and most especially after the initial surgical examination of the wound or the necessary debridement’.^19^ In the years that followed the Rif War and until the outbreak of the Spanish Civil War, Bastos would have reached ‘among us [in Spain] the maximum perfection’^20^ in the application of the procedure.

Despite these claims, it is still unclear today, on the one hand, what the exact technique used by Bastos to treat the wounded in Morocco was, when he began to use it, how many fractures and of what type he treated, and where he disseminated his findings. These shortcomings have been justified on the grounds of ‘the scarcity of Bastos’ writings on the Spanish colonial wars’.^21^ Although not a prolific and data-rich author, he nevertheless did publish regularly and provide statistical evidence. On the other hand, historiography has not sorted out whether Bastos developed his own autonomous version of the closed method or relied on the contributions of other Spanish and foreign surgeons, Orr in particular. This is a key question for whose analysis all historians have hitherto relied on his autobiography *De las guerras coloniales a la Guerra Civil. Memorias de un cirujano* [From the colonial wars to the Spanish Civil War. Memories of a surgeon] (henceforth, *Memorias*). Bastos claimed there categorically that

this [closed] method - which I might well call *my* method, though I don’t - was adopted by all Spanish surgeons on my side [Republican] in the hecatomb of our Civil War. It was later taken to England by Dr. Trueta and spread all over the world under the name of ‘Spanish method of occlusive cure’. But its authorship [Bastos’] has been recognised and proclaimed by the said doctor [Trueta] in his well-publicised publications, as well as by all those who have dealt with this aspect of war surgery. I do not intend to show off, just to leave a record of what happened.^22^

A superb piece of writing, Bastos’ *Memorias* are, however (as this type of historical source in general is), a much belated account of events and, as such, liable not only to accidental and deliberate omissions, but also to reinterpretations resulting from both the author’s own biographical evolution and the new historical and social circumstances from which the text is written.^23^ Regarding the latter, Bastos published his memoirs during General Francisco Franco’s dictatorship, which, despite the relative liberalisation of the late 1960s, still precluded or restricted mentions to certain actors, institutions, and events of decisive importance in his professional career. Similarly, international medicine had undergone profound transformations since the days preceding the First World War, making it very hard to acknowledge the weight that certain ideas and practices, as well as individuals, groups, or countries had had in earlier periods. Based on these and previous considerations, the main aim of this article is to provide a more solid and situated reconstruction of Manuel Bastos’ early contributions to the development of the closed method, especially his practice during the Rif War. For this purpose, it will be necessary to explore how exchanges between military and civilian, wartime and peacetime surgical practices took place in Spain. It will also be necessary to unearth neglected trans-Atlantic circulations between Spain, France, Latin America, and the United States during the interwar period, embodied mainly in the figure of the Argentinian surgeon Pedro Chutró. Ultimately, this paper is an attempt to re-centre^24^ the story of the closed method.

## Bones of soldiers, bones of children

**Figure 2.**
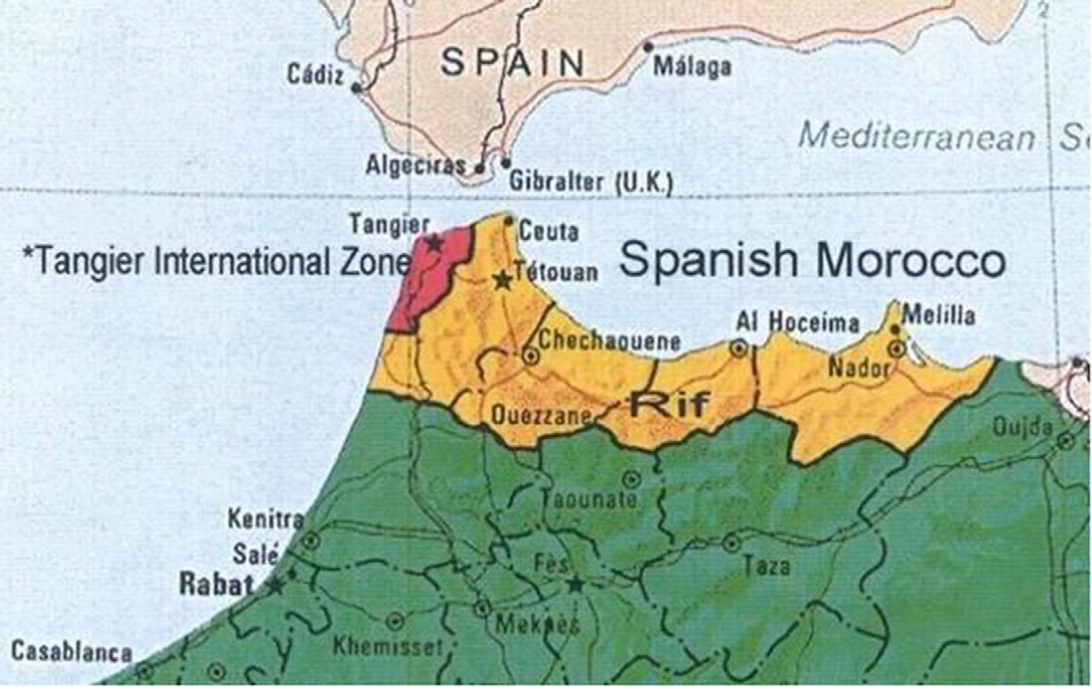
Territory of ‘Spanish Morocco’ (in orange) during the Protectorate period 1912–56. Source: Perry-Castañeda map collection, University of Texas Libraries, University of Texas at Austin. Public domain. Figure 2. long description.

The early orientation of Manuel Bastos towards orthopaedic surgery and traumatology – the latter speciality, as Roger Cooter has shown, only slowly emerging from general surgery between the late 19th century and the 1930s^25^ – took place in a dual professional context. On the one hand, Bastos joined the Army Medical Corps just months after graduating in Medicine in Zaragoza in June 1907. A son of a military officer, he knew that army physicians were the largest group of state doctors in Spain, while civilian practitioners suffered from administrative atomisation and financial insecurity except for the case of the Marine Health Corps (*Sanidad Exterior*).^26^ Following the compulsory one-year special training at the Army Medical Academy in Madrid, Bastos was assigned to various posts in which he gained his first practical experience as a surgeon. The most important were those in the Spanish Protectorate in Morocco, a tiny enclave adjacent to the Strait of Gibraltar which Spain had begun to occupy some years before its official establishment in November 1912 (Figure 2). Bastos took part in the military operations that followed a surprise attack by Riffian (Amazigh/Berber populations of the Rif mountain range) insurgents near Melilla in September 1909 that killed 150 soldiers and wounded another 600.^27^ Over 30,000 Spanish troops were shipped across the Strait to retake the lost territory, though the vast majority of military actions in the Protectorate prior to 1921 involved much smaller contingents. As the Riffians, at this stage, were only equipped with Mauser rifles, lacking artillery, gunshot wounds and fractures were neither frequent nor too serious.^28^ Bastos grew increasingly familiar with their treatment and evacuation, as for example in a clash near the iron mines in the Melilla region, in which he assisted two hundred wounded on the spot and took them over to that town.^29^

He may also have learnt innovations in the management of compound fractures that senior colleagues were pioneering. Captain doctor Sebastián Lazo, director of a civil-military dispensary near Melilla, advocated for ‘the method that has come to be called *Japanese* [as developed in the Russo-Japanese War of 1904-05]’, which had achieved a ‘very low mortality rate in our wounded soldiers’. This method consisted of avoiding any cleansing of the wound with antiseptic solutions and any examination beyond the indispensable to confirm the existence of a fracture; to disinfect the wound orifices with iodine, dress it with aseptic gauze, cover the area with a simple aseptic bandage, and ensure that the patient stood in ‘complete rest’.^30^ More specifically, in relation to limb fractures, whose frequency had been on the rise since the days of the Second Boer War (1899–1902) due to developments in weapons and projectiles,^31^ captain doctor Rafael Ramírez-Rivas, also stationed in the Melilla area, pointed to the ‘paradoxical fact that more and better consolidations are achieved in cases in which these [forearm] fractures are open and the surgeon can therefore directly act upon the focus of the fracture, as often happens in war surgery’.^32^ Ramírez-Rivas claimed he knew of a hundred cases in which the wounded ‘have not lost their limbs, the [open] fracture consolidating with greater perfection than in cases of simple fracture’, and re-joined the ranks ‘to finish their duty for the country […] preserving intact, or just slightly diminished, the forearm’s pronation and supination’.^33^

In addition to technical advances, it became clear to Bastos how important it was to ensure a good evacuation from the front line so that further damage was prevented, especially in the abrupt mountainous orography of northern Morocco. During his second assignment in the Protectorate in 1913, he operated two hundred men wounded in a battle at Laucien (19 km east of Tetouan). Two days later, he transported them to the military hospital of that city and

continued to assist them personally […] until the doctor who had to replace me in the battalion showed up. I was then free to return to Spain, but I did so at the head of an expedition of wounded who were taken by ambulance [from Tetouan] to Ceuta, from there by boat to Algeciras, and from there by hospital train to Madrid. They were those who had received my first aid at the front. I was thus able to supervise them in the most delicate stage of their recovery, and I sincerely believe that this was of great benefit to them. All the more so because I was able to continue to care for many of them until they were completely cured.^34^

Bastos became a fracture specialist in a civilian context too. In the early twentieth century, Spanish army doctors were allowed to combine this work with positions in the general health system and administration, in the university, or in private medicine. Their action also reached well beyond military personnel through the provision of outpatient and hospital care to their families, to the general population of certain small towns or peripheral regions, and in case of major epidemics.^35^ This militarization of Spanish medicine and public health just mirrored that of Spanish politics and society. On returning in 1910 from his first assignment in Morocco, Bastos began his civilian trajectory at the *Instituto de Terapéutica Operatoria* [Surgical Therapy Institute] or *Instituto Rubio* in Madrid. This was a private teaching and assistance centre founded by Federico Rubio Galí, a renowned physician, hygienist, and politician who decisively contributed to the introduction of many health innovations in Spain.^36^ Rubio’s activities were inspired by and connected with the ‘regenerationist’ drive that since the end of the nineteenth century struggled to modernise the Spanish administration, economy, and education under the guidance of organisations such as the *Institución Libre de Enseñanza* [Free Teaching Institution].^37^ Bastos entered the *Instituto*’s department of Orthopaedic Surgery, directed by Adolfo López-Durán (1877–1930). Although mentioned several times in his *Memorias*, these failed to reflect the professional and scientific weight of a relation that would last for two decades. López-Durán had achieved a solid position in Madrid’s medical milieu under the protection of the surgeon and politician Alejandro San Martín.^38^ In addition to the *Instituto Rubio*, he also worked as a consultant at the Royal Palace, head of a service at the Provincial Hospital, and inspector at the National Maritime Sanatorium of Oza (Galicia) for children with tuberculosis. His main specialisation was precisely the surgical treatment of bone lesions caused by that disease, then rampant among Spain’ working class, as occurred in most Western countries. López-Durán published many articles and several monographs, some of which circulated abroad.^39^ He presented his research mostly at national conferences such as the 3rd Spanish Congress on Tuberculosis held in San Sebastian in 1912 or the 2nd National Congress of Medicine in Seville in 1924. With the experience gained in two years of work with López-Durán, Bastos prepared his 1912 doctoral thesis *Anatomía y mecánica de la bóveda plantar y sus deformaciones* [Anatomy and mechanics of the arch of the foot and its deformities]. This study aimed to provide a solid structural basis for the handling of bone lesions caused by trauma or disease.

**Figure 3.**
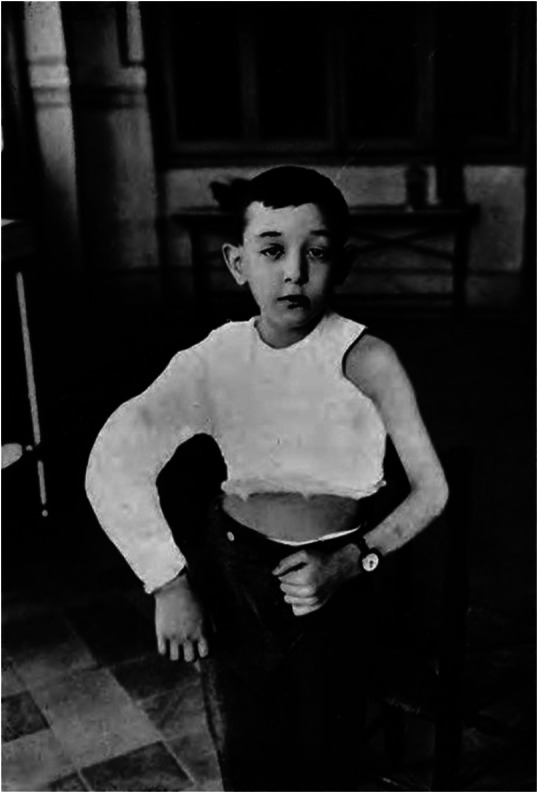
‘Containment device’ for humerus fracture in a child. Source: López Durán, Bastos Ansart, 1915, 295. Public domain.

After his second stay in Morocco, Bastos took back his research with López-Durán, though this time with a clinical-surgical approach, probably influenced by his military experience. The main outcome was a joint monograph with the revealing title *La práctica del tratamiento de las fracturas de los miembros* [Praxis of the treatment of limb fractures] (1915), which constituted Bastos’ debut into fracture therapy. It was based on patients assisted at the *Instituto Rubio* and the *Clínica Militar de Emergencia* [Military Emergency Hospital] (an antenna of the suburban Military Hospital of Madrid-Carabanchel in the centre of the Spanish capital). A chapter of this book, ‘Treatment of fractures of the upper section of the humerus’, was published in advance as a journal article by the end of 1914. The authors did not explain why they chose these fractures, and not others, for an independent publication, but we believe this resulted from the convergence between López-Durán’s interest in the increasing number of closed fractures of that bone in tuberculosis patients he treated in Madrid and Galicia, and Bastos’ interest in the increasing number of open fractures of the arm in soldiers wounded in Morocco. In their article-chapter, López-Durán and Bastos presented a ‘containment device’ (Figure 3) of their own as the most appropriate means of treating closed humerus fractures in children. Given the characteristic ‘restlessness’ of these young patients and the difficulty of keeping them bedridden for long periods of time, the best way to ensure immobility was to use a plaster bandage. This was prepared by placing a layer of cotton wool over the chest, shoulder and affected limb of the child and then covering it with ‘circular wraps of plaster bandages […] which have been dipped in water heavily charged with alum, squeezing them well so that they set quickly, and in such a way that they encircle the chest, shoulder and arm up to the wrist’.^40^ This device was the first move towards the practice of the closed method we have been able to identify in Bastos’ career.

In fact, López-Durán and Bastos considered plaster bandages to be the most useful treatment for humerus fractures in adults too. Unlike splints, which ‘immobilise, but do not achieve a well-reduced containment of the fragments’, therefore being regarded mainly useful for first aid cures and evacuation, plaster bandages ‘can hold the fragments better […], provided they are used […] in those cases in which the reduction has been achieved in a perfect way’.^41^ Quoting the French surgeon Just-Lucas Championnière, head of service at the Hôtel-Dieu in Paris and author of the influential *Précis du traitement des fractures par le massage et la mobilisation* (1910), they concluded that

immobility not only attenuates or suppresses pain and inflammation, but ‘is the best way to promote the repair of fractures’. Indeed, it has been proven that strictly immobilised fractures heal earlier and better than those allowed some mobility, even if very little. […] In our opinion, this method should be used for all fractures of the upper limb […].^42^

## The making of Orr’s and Bastos’ methods

López-Durán and Bastos’ decision to advance the publication of that chapter of his book was also influenced by the recent outbreak of the First World War in July 1914. In addition to over 20 million dead, this conflict resulted in hundreds of thousands of cases of open gunshot wounds caused by machine guns, mines, artillery shells, and airplane bombs.^43^ In all belligerent armies, doctors struggled to improve the management of these patients. Hiram Winnet Orr did it in successive assignments in England, Wales, and France from May 1917 to June 1919, where he began to use the plaster bandages that would later become a distinctive feature of the closed method. At his first post in the Whitechurch Hospital of Cardiff, he observed ‘the unhappy results of treatment and the long delay in healing that followed many cases of open fractures resulting from gunshot wounds’,^44^ concluding that successive wound dressings were a negative factor in the evolution of patients. Orr was later transferred to Base Hospital No. 8 in Savenay, France, which served as an evacuation centre for all US Army base hospitals in that country, sending ‘large numbers of wounded […] to the United States’.^45^ These wounded, up to 5,000 a month, were distributed at the moment of arrival in separate wards for femur fractures (192 beds), arm fractures (256 beds), leg fractures (128 beds), knee joint injuries (34 beds), or amputations (320 beds).^46^ They were then prepared for the long transatlantic trip with splints so that the damage caused by the ships’ rocking was neutralised. Only when the supply of splints ran out and despite opposition from colleagues and chiefs, Orr ‘obtained permission to send home patients with open fractures encased in plaster, even though suppurating wounds were present’.^47^ The goal was then to prevent further damage in compound fractures, not to heal them, so

*large windows were cut over all open wounds* and over Patellae, heels, etc. Casts were not split. Plaster of Paris was used especially for fractures of the femur, leg, and upper arm. In femur and upper arm cases, body casts were employed. From September to December [1918] about one thousand plaster casts per month were put on and practically all sent to the United States. No complications as to casts were reported and in general the patients were found to have travelled safely and comfortably.^48^

Orr’s case seems to confirm Leo van Bergen’s claim that medical innovations are not as frequent in wartime as often implied, due, among other things, to the priority of military doctors being the return of the wounded to the ranks rather than their cure, and to the difficulty of carrying out a long-term follow-up.^49^ It was only after the war, with his return to civilian practice in Nebraska, that the American surgeon made plaster bandages the basis for a treatment of open fractures of the limb that prevented the development of chronic osteomyelitis of the type he frequently observed in industrial accidents’ victims. Orr first presented his ‘closed method’ in two articles of February 1923 and November 1924. In the former, published in *The Nebraska State Medical Journal*, he gave a full, systematic description of the technique. Building on a general ‘principle of rest’, Orr prescribed the ‘restoration of the injured parts to normal relationship’ and the wounded limb’s ‘immobilization and protection in correct position until complete healing has occurred’.^50^ In between these two steps, the surgeon should proceed ‘to “saucerizing” both soft tissue and bone as suggested by [Alexis] Carrell, [Pedro] Chutró, and others’; to cleaning up the wound as thoroughly as possible; so that finally ‘the saucer-like opening of both soft tissues and bone is gently packed with sterile Vaseline and gauze’.^51^ Once this was done, the whole area should be covered with dry sterile gauze and the entire limb ‘encased in a well-fitting cast of sufficient extent to completely immobilize the infected part. Casts are not split, and *no windows are cut.* If there is no indication on account of a rise of temperature for a change in dressing, this dressing is left in place for ten days to two or three weeks’.^52^ Orr claimed to have applied this method to 25–30 patients ‘with much better results than formerly and without danger’.^53^ His second, 1924, article contained only a short description of the technique, though it reached a much larger public thanks to its being published in the *Journal of the American Medical Association* and in collaboration with this prestigious society’s vice-president, James Edwin Thomson. Orr and Thomson explained that

in the employment of our technic for the treatment of compound fracture wounds, which we have fully described elsewhere [1923 article], the wound is cleaned up or debrided, filled lightly with a petrolatum gauze pack, and is covered under the cast with a dry, sterile pad. […] Unless there is marked rise in temperature, the wound remains untouched for from two to six weeks, at which time a window is made over the wound, and the first dressing is removed. […] Our experience in treating fractures by this method is that the period of healing is only a little longer than that of simple fractures, and the ultimate function of the limb is much more satisfactory than by other methods we have employed.^54^

The closed method would be the object of new articles by Orr in 1927 and 1928 and of the 1929 treatise *Osteomyelitis and Compound Fractures and Other Infected Wounds* ^55^. In the Anglo-Saxon world, they enjoyed a wide circulation, though with mild success. In other regions, the situation was more complex. In the case of Spain, which did not participate as a belligerent in the First World War, army surgeons had missed the experience with fracture treatment of their European and US colleagues. It has been assumed that this gap was only closed with the outbreak of the Spanish Civil War in 1936. However, the so-called Rif War (1921–27) provided an earlier chance for catching up with world war innovations. This conflict, still badly known today, brought a significant escalation in Spanish operations in Morocco in relation to the previous decade, both in the size of the contingent involved, in the type of armament (heavy artillery, tanks, planes, and chemical weapons), and in the number of dead, wounded, and sick soldiers.^56^ As a result, the Army Medical Service became rapidly overwhelmed in the Protectorate, Ceuta, and Melilla, and the peninsular military hospital network was flooded with thousands of cases of gunshot wounds, malarial fevers, tuberculosis, and other ailments.^57^ Manuel Bastos returned then to active service from a long leave of absence and rushed to assume the management of limb fractures as director of the ‘fracture centre’^58^ that functioned at the newly established Malaga Base Hospital between September 1921 and August 1922. That centre treated the wounded who had been evacuated in hospital ships from all over the Protectorate territory after receiving first aid care at the front and further assistance in the rear-guard hospitals of Melilla, Ceuta, Tetouan, and Larache. After the latter date, Bastos moved back to the Military Hospital of Madrid-Carabanchel, where he continued to operate fractures with his ‘surgical team’ while running a clinic for the ‘re-education of war wounded’.^59^

In his *Memorias*, Bastos claimed to have used the closed method during the Rif War. He affirmed that in a paper published at that time he had given a detailed account of ‘my method’ of treatment with plaster dressings, which was applicable not only to simple fractures, but also to those complicated with wounds. The procedure’s novelty

lay mainly in applying it to the latter as if they were closed, that is, by immobilising the limb in plaster and not worrying about the wound, which was walled up in the dressing and deprived of any access from the outside. In those days this looked reckless. We did it with great apprehension and only in cases of linear path [gunshot] wounds. But as we saw that these healed well with the occlusive cure [*cura oclusiva*] regime, we proceeded to apply it systematically.^60^

**Figure 4.**
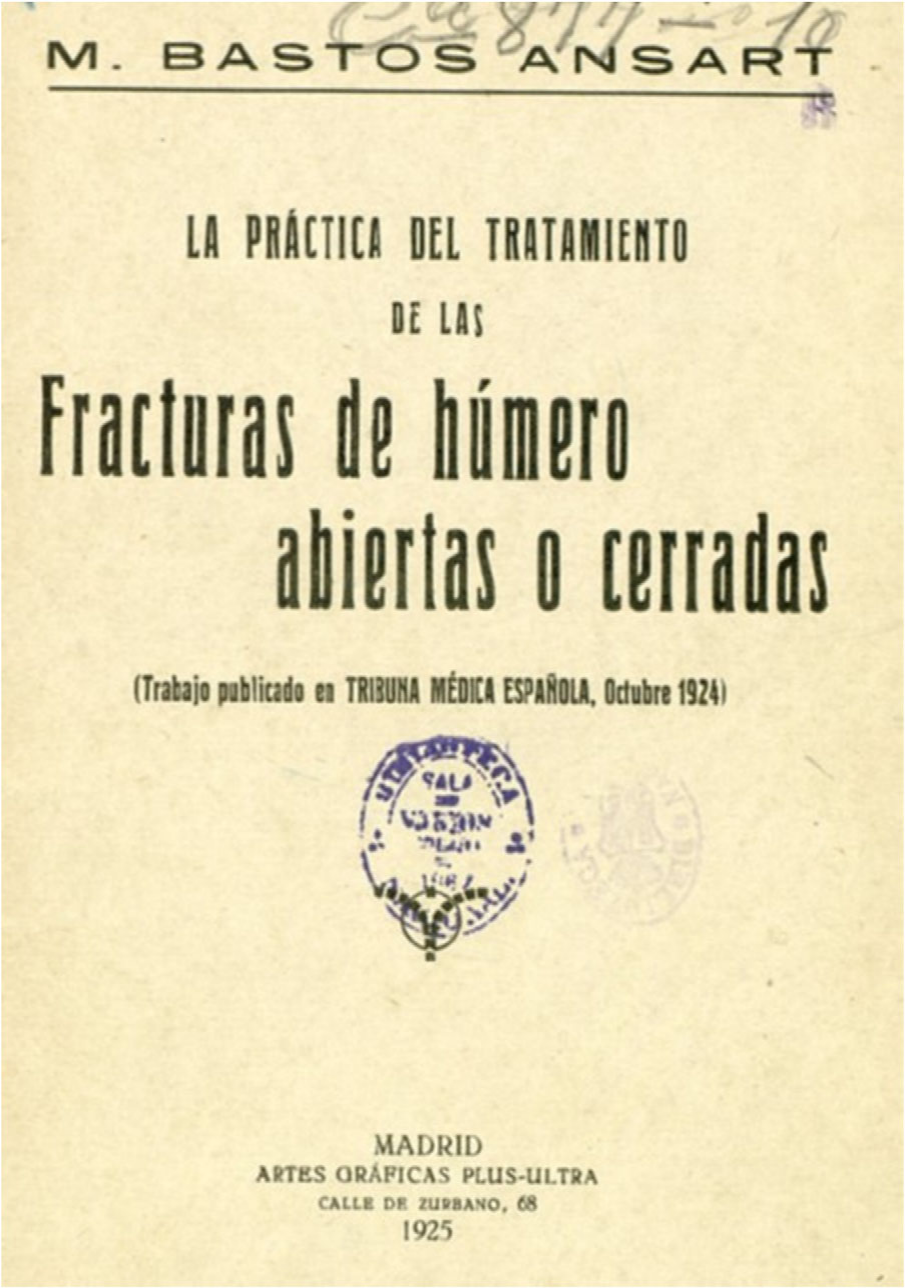
Front cover of Bastos’ offprint of his paper on humerus fractures. Source: Bastos, 1925. Imágenes procedentes de los fondos de la Biblioteca Nacional de España.

The article to which Bastos referred, *La práctica del tratamiento de las fracturas de húmero abiertas y cerradas* [Praxis of the treatment of open and closed humerus fractures], was published in October 1924 in the first issue of *Tribuna Médica Española* [Spanish Medical Tribune] (henceforth, *Tribuna*).^61^ The restricted circulation of this journal led him to republish, a few months later, an offprint of the article (Figure 4). This may explain why specialists in Bastos have barely used it in their research. Another reason is that the Spanish surgeon did not coin a particular term for the technique that Orr had just christened ‘closed method’ (the equivalent Spanish term of ‘*cura oclusiva*’ only appeared in his 1969 *Memorias*). The paper presented, nevertheless, results of the application of ‘his’ method (as well as of various other treatments: osteosynthesis with Lambotte plates and Parham-Putti-Lambotte bands; suspension of the arm with irrigation and cures). Once again, the election of the humerus revealed the sustained interest of Bastos in this bone. After ten years of work and research, his approach was based on the belief that

it is very rare that a fracture of the humerus becomes life-threatening. Infection is never very serious […], probably because of the thinness of the soft tissues and the absence of strong aponeurotic septa which, in other limb segments allow infection to run along the muscle packages reaching a long distance away from the wound. From this point of view, a war fracture of the forearm is much more serious than a fracture of the arm.^62^

**Figure 5.**
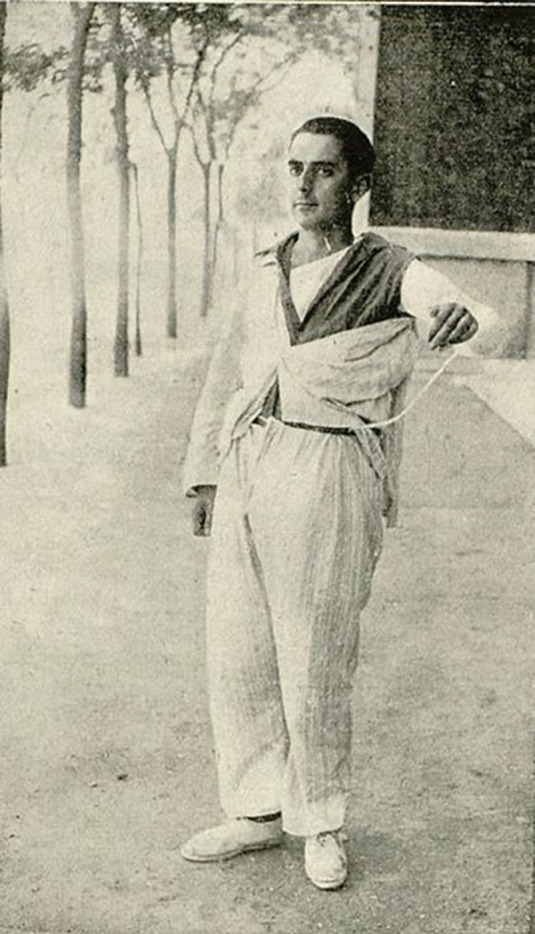
‘Abduction device’ for humerus fracture in a Rif War wounded soldier. Source: Bastos, 1925, 18. Imágenes procedentes de los fondos de la Biblioteca Nacional de España.

The treatment of these fractures was thus reduced for Bastos essentially to a problem of consolidation, i.e. how to ensure a good callus formation and avoid pseudarthrosis. His surgical practice began with a restricted use of the Friedrich technique, for these fractures seldom gave reason for an intervention to clean the focus; when they exceptionally did, it was necessary to ‘restrict oneself to the ample opening of the soft parts, removing from the focus only the fragments that were absolutely loose and displaced in the soft parts or in the medullary cavity’.^63^ A second step consisted of a rigorous immobilisation, ‘not just restricted to holding the arm, but keeping the shoulder and elbow absolutely motionless’, to the point that reduction became secondary as long as the arm ‘was placed in a physiological posture of relaxation of all the muscles’.^64^ Bastos achieved this posture, which for all fractures of the humerus ‘is abduction of 45°, more or less, with flexion of the forearm at right angles and half rotation (i.e. the arm remaining at the level of the nipples)’, by means of an ‘abduction device’ (Figure 5) of his own design. It consisted of a circular cast stretching from the shoulder to the lower third of the forearm and leaning ‘on an iron [rod] with a fork in each extreme: the thin one is embedded into the arm cast, the large one, into a sort of cast belt that serves as support for the abduction bar [the iron rod] so that its weight does not rest on a single point of the waist’.^65^ Immobilisation lasted four weeks on average, until radiographic evidence of a relatively strong callus was obtained,^66^ after which the cast was replaced by a ‘tighter one, from armpit to wrist, but without abduction’ for another month, and then by a splint one last month. Despite the prolonged fixation, he never observed ‘any stiffness or limitations of movement of the elbow’ following the treatment.^67^ In all cases, except in one resulting in pseudarthrosis ‘due to extensive loss of substance’, he achieved ‘a good consolidation with functional integrity’,^68^ even in some in which the humerus was ‘disintegrated into innumerable fragments, which, nevertheless, later appeared all to be enclosed in a normal callus’.^69^

The *Tribuna* article reported the results of the application of this procedure in about 50 cases of open fracture of the humerus ‘and almost as many of closed fracture’. That number was substantially higher than in Orr’s 1923 paper, but Bastos admitted that, in many of them, the plaster had been fenestrated for small wounds while for larger ones the whole area had been ‘left exposed to the air’ with the plaster covering only elbow and forearm.^70^ This is consistent with the Spanish surgeon’s ‘great apprehension’ towards the new technique. Nevertheless, the fewer cases in which Bastos had truly used a closed method and for which he had gathered ‘sufficient data on their evolutive course […] from 1921’^71^ paved the way for a more systematic use of the technique in later stages of the conflict, when fresh large shipments of wounded soldiers arrived at the Madrid-Carabanchel Hospital following the Chechaouene retreat of late 1924, the Al-Hoceima Landing of September 1925 and the final offensive of May 1926 that led to the surrender of the Riffian leader Muḥammad bin ‘Abd al-Karīm al-Khaṭṭābī. Although Bastos did not publish other papers on the subject, and we have hitherto failed to find clinical records or statistics in military archives, it seems plausible to think that the more extensive use of the closed method by Bastos and his team took place in those final stages of the Rif War.

The ‘abduction device’ with which Manuel Bastos treated open humerus fractures in the Moroccan war wounded externally resembled the ‘containment device’ designed in 1914 with Adolfo López-Durán for humerus fractures in children. There were, however, significant differences between them resulting from a different understanding of those fractures and a new way of applying the plaster cast. Regarding the former, López-Durán and Bastos had originally advocated for an early mobilisation of the arm by active movements of the patient, or else by massage and manipulation. Besides, the posture of the limb determined by the containment device, without being so detrimental as the one set by the much criticised ‘airplanes’ that became popular during the First World War, provoked an excessive degree of separation and elevation with respect to the trunk. Regarding the plaster cast, they had not initially applied it directly on the skin and bony reliefs, but over a gauze ‘pad’, with the consequence that it moved because ‘it rests more on certain points than on others, [and it] deflects or changes its position because the cotton layer is crushed and the infiltration of the fractured limb decreases, so this takes on an unequal weight which alters the position of the bones’.^72^

This significant evolution in Bastos’ surgical practice owed much, for sure, to his intellectual skills and professional experiences. But, as just shown, it ran parallel to the work of other international surgeons whose war and post-war contributions he may have been acquainted with. The most outstanding was Orr. We believe, however, that Bastos ignored the method proposed by the American surgeon in his 1923 and 1924 papers when he treated his first Rif War patients and published his *Tribuna* article. The Spanish surgeon admitted ‘following Orr’s prescriptions’^73^ in his classic 1936 treatise *Algunos aspectos clínicos de las heridas por arma de fuego* [Some clinical aspects of gunshot wounds] but did not clarify when exactly he had become a follower. Everything suggests that the American surgeon’s impact in Spain only began with his *JAMA* article of November 1924. Joaquín d’Harcourt, one of Bastos’ favourite disciples in Madrid, claimed in a 1939 article that ‘*on ne trouve rien, dans la littérature médicale moderne, jusqu’en 1924, moment où le chirurgien nord-américain Orr préconise la cure occlusive dans les ostéomyélites chroniques*’ [nothing can be found in modern medical literature until 1924, when the north-American surgeon Orr advocated the closed method as treatment for chronic osteomyelitis].^74^ Josep Trueta, who represented the parallel Barcelona tradition in orthopaedic surgery and traumatology, agreed with D’Harcourt when he pointed out, in his posthumous memoirs, that ‘in 1924 there appeared an article by a North American surgeon, Winnett Orr, from Nebraska, which recommended a new technique that allowed patients to be sent home sooner than with any other treatment’.^75^

The fact that Orr’s *JAMA* article appeared a month after Bastos’ paper makes it impossible that the latter used it. As for Orr’s 1923 article, it is highly unlikely that Bastos knew of it, given the peripheral circulation, even within the United States, of *The Nebraska State Medical Journal.* The last option left for Orr’s influence upon Bastos would be another *JAMA* article published in July 1922. However, no mention or description of the closed method was made there, not even briefly. Orr just made this short comment on the treatment of open fractures:

My principal point […] is that neither the Carrel-Dakin method nor any other kind of wound treatment should be permitted to interfere seriously with the fundamental principles in the treatment of bone and joint injuries. Those principles are, first, the immediate replacement, as nearly as possible, of injured parts in anatomic relationship, and second, the maintenance of immobilization […] of such injured parts in proper relationship until healing occurs.^76^

## The rise of Pedro Chutró and his impact on Bastos

**Figure 6.**
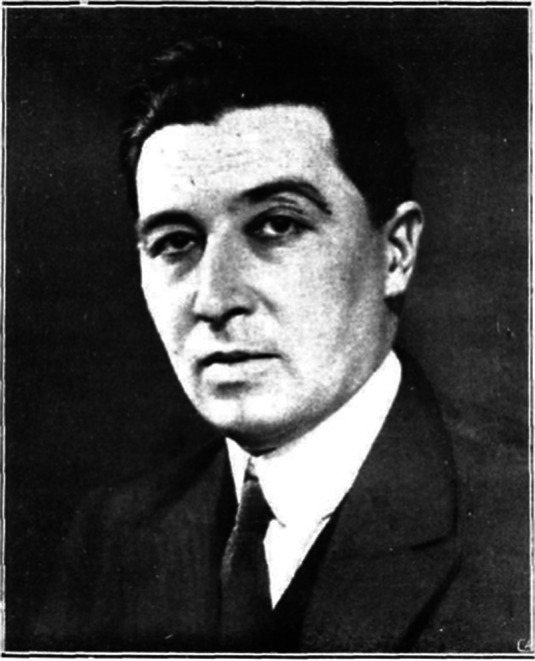
A portrait of Dr. Pedro Chutró. Source: Mundo Gráfico, 15 October 1924. Imágenes procedentes de los fondos de la Biblioteca Nacional de España.

It seems to us highly improbable that Bastos knew of Orr’s method during the early phase of the Rif War. However, a parallel version of the technique may have reached him before the outbreak of that conflict through the nowadays forgotten Argentinian surgeon Pedro Chutró y Cortejarana (Pila, 1880–Buenos Aires, 1938) (Figure 6). Like López-Durán, Chutró received less attention than he deserved in Bastos’ *Memorias*, though his importance for the Spanish surgeon can be hinted at in the following statement:

Also assiduously frequenting our clinic [at the Madrid-Carabanchel Hospital] was Professor Chutró, from Buenos Aires, who had achieved universal fame for his surgical work with the allied troops during the last great war. His lectures in various scientific and cultural centres were a great success and left a deep impression. They were all brimming with original ideas, full of good sense and profoundly innovative […] We are doing today much of what Chutró told us, though we no longer remember who taught it to us.^77^

Bastos still remembered in 1969 the time ‘when the Argentinean country, so closely linked to ours, was in full splendour and at the height of its world prestige’, but most had forgotten it by then. The Nobel Prize won by Bernardo Houssay in 1947 had represented the culmination of decades of development of health innovations and biomedical research in Argentina, as well as of intense exchanges with the most advanced countries in America and Europe.^78^ Spanish-Argentine circulations, in particular, boomed in the early 20^th^ century following the renewed ‘reunion of Spain with America’ after living with their backs on each other for much of the 1800s.^79^ This reunion became especially close during General Miguel Primo de Rivera’s Dictatorship (1923–30), for this took Hispano-Americanism^80^ and *Hispanidad* ^81^ as foreign policy hallmarks. Perhaps the most significant example of the extent of Spanish-Argentinian medical relations at that time was the 2nd National Congress of Medical Sciences held in Seville on 15–20 October 1924. So many Argentinians registered and/or participated^82^ that 14 out of the 15 panels had specific sub-sections for presentations by Argentinian doctors.^83^

Chutró’s influence in Spain and other countries resulted from his prominent role in the First World War. With a French family background, he felt called to volunteer for France during the conflict, getting in contact with Antonin Gosset, head of the surgical service of the Pitié-Salpêtrière Hospital in Paris, with whom he had already worked in 1906.^84^ At the beginning of the war, Gosset was appointed director of several military hospitals in the French capital, including the one established at the Lycée Buffon with more than 300 beds.^85^ When Gosset was eventually assigned to the front, Chutró took over the direction of this hospital. He would hold it from June 1915 to April 1919^86^, what gives a measure of his prestige and of the trust French authorities placed in him, as did his admission at the *Société de Chirurgie de Paris^^87^^* and his decoration with the *Legion d’Honneur.* During the war, Paris became a global crossroads of medical figures, ideas, and practices.^88^ Numerous innovations emerged, more or less successful, as a result of these transnational circulations. In the case of the Lycée Buffon hospital, these ranged from the disinfection of wounds with the Dakin-Carrel solution, to new gas gangrene treatments, to blood transfusions, all of which spread internationally because Gosset hosted many foreign surgeons such as Chutró, the Spaniards José de Sard and Mariano Gómez Ulla, the Brazilian Mauricio Gudin or the Russian (later naturalised French) Arnault Tzank.^89^ The fact that the Lycée building also housed the American Base Hospital in Paris enabled Chutró to meet US surgeons who worked there or visited the centre. According to the US government order awarding him the ‘distinguished service medal’ in July 1918, he had

devoted himself unreservedly to teaching American medical officers the principles of the lessons learned through experience by the French and British surgeons in the first years of the war, with the result that the knowledge so imparted assisted in a great measure to conserve the life and limb of thousands of American and allied wounded.^90^

Among his American visitors, shortly after the signing of the armistice, was Orr himself. In his memoirs *An Orthopedic Surgeon’s Story of the Great War*, the US surgeon recalled that he was

[…] ordered to Paris [from Savenay] on several occasions to attend the Red Cross Hospital Conferences, and to see the work of the Base hospitals in that area. It was upon one of these visits that I had the privilege of seeing the work of Professor Chutró. He had worked out a unique organisation and an operating room and dressing technique. His service was the only absolute surgical monarchy I have ever seen. But he was doing fine surgical work and securing some excellent results.^91^

The Argentinian always requested ‘the worst cases’,^92^ yet his approach based on restricted surgical interventionism was consistently well appreciated by Orr and other Allied surgeons.^93^A British commission noted that the dressing of wounds was renewed ‘in his hospital service only about once a week […], no bacteria chart is kept […] smears are examined only once in ten days’, and no surgical closure of infected wounds was made. In general, Chutró was not dependent upon a definite assurance that a wound was ‘clinically sound’, being satisfied if there was no suppuration, if it steadily healed, ‘and if the patient’s general condition is good and the temperature normal’.^94^ More specifically in relation to fractures of the humerus, which he discussed at a session of the *Société de Chirurgie* in 1918, Chutró considered that the abundance of pseudarthrosis was often due to excessive intervention by the surgeon, either because of ‘an early, too large bone debridement’ which resulted in the loss of bone and periosteal substance, or because of ‘the application of containment devices causing an exaggerated exacerbation of the fragments and thus favouring fibrous interposition’.^95^ Chutró rejected such procedures, advocated among others by the French surgeon Pierre Delbet, preferring immobilisation in plaster even if it ‘would not guarantee the strict reduction of fractures’.^96^

In the immediate post-war, Chutró, like Orr, paid great attention to the problem of chronic osteomyelitis (he preferred the term ‘bone fistula’ for those resulting from war wounds, leaving the other name for those of ‘civilian’ origin). It was precisely because of his prestige in this subject that at the close of the war he was ‘requested to go to New York to demonstrate in the military hospital there his original methods of dealing with old, infected compound fractures with osteomyelitis’.^97^ During his stay, he gave a three-month course at the New York Polyclinic,^98^ a private medical school for postgraduates founded in 1882,^99^ and presented a paper at the 70th Congress of the American Medical Association (AMA) held in Atlantic City in June 1919, published shortly afterwards in *JAMA* under the title ‘Bone fistulas after war wounds’.^100^ For all these reasons, US military surgeons cited him as an authority on a disease which

has followed in the wake of all wars, but the use of high explosives and the machine gun in this war, with a consequent greater number of bone injuries, has increased proportionately its occurrence. […] Indeed, the number of its victims shakes our confidence in what we had come to believe of modern war surgery. Dr Pedro Chutró, of Buenos Aires, who has been in France since the beginning of the war, and who is recognized as one of the foremost of war surgeons, reports that there were 75,000 old suppurating bone sinuses [fistulas] in France alone at the close of 1917, the third year of the use of the Carrel-Dakin method (Chutró lately reports that there were 300,000 of these cases in France at the end of the war).^101^

Orr attended the congress too, presenting a paper in the Orthopaedic Surgery section.^102^ It would be of great interest to investigate whether this new encounter led to any personal or intellectual exchange between them or had any impact on the US surgeon’s subsequent research and publications on the closed method. In any case, Chutró returned to Buenos Aires, where he took over the chair of Surgery from his mentor Diógenes Decoud before taking on a ‘study trip’ to Europe in late 1920. During this new stay in France, he would be appointed foreign correspondent of the *Académie de Médecine*, thereby deepening his lasting professional connection with this country in which he was held in the highest esteem.^103^

Chutró’s ideas on fractures and osteomyelitis reached Spain during the First World War through Spanish doctors who worked with him or visited his service. A prominent example was José de Sard, who spent several months with Chutró at the Lycée Buffon hospital. In a long lecture on the medical advances of the war at the 1st National Congress of Medical Sciences held in Madrid in 1919, Sard explained that in the treatment of femur and tibia fractures he had obtained ‘good results with the Finochietto device, introduced in France by my friend Chutró’.^104^ The latter actually used it to achieve continuous traction of the limb, not temporary as Finochietto, and wrapped the metal tape (which crossed the heel above the calcaneus and behind the Achilles tendon) and the stirrup (from which the weight that made the traction hung) inside the plaster bandage, protecting both with ‘a voluminous cotton and gauze dressing, which acts as a bacterial filter’.^105^ Bastos, who presented two papers at the congress, must have attended Sard’s lecture.^106^ Perhaps then or after a visit to Paris in 1920,^107^ he became interested in Chutró’s research. He may have learned that the Argentinian surgeon had devoted his 1904 doctoral thesis to the fractures of the upper segment of the humerus in children, a subject very similar to that of his first publication with López-Durán in 1914. And also that during the war he had approached the problem of pseudarthrosis of the humerus from a deep knowledge of the anatomy of that limb, close to his own early ‘kinematic’ approach to fractures.

**Figure 7.**
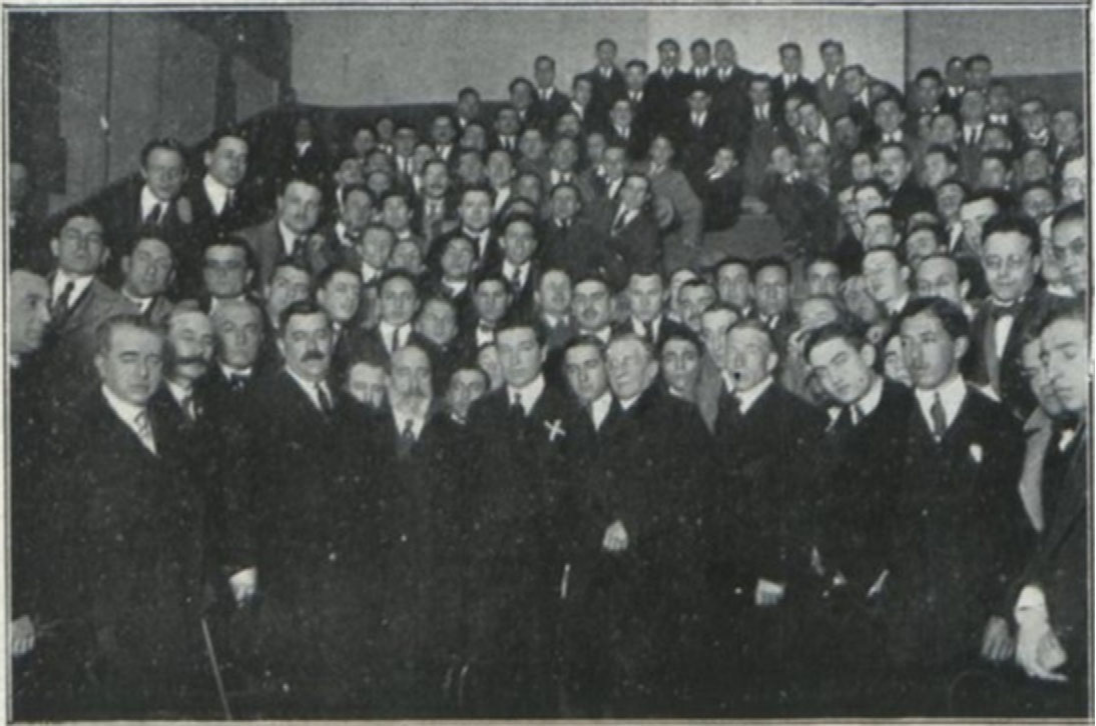
Pedro Chutró (marked with an X) during his visit to the Faculty of Medicine of Madrid in 1921. Source: La Hormiga de Oro, 26 February 1921. Imágenes procedentes de los fondos de la Biblioteca Nacional de España.

Chutró’s impact on Spain’s surgery, and on Bastos in particular, was boosted by his first visit to this country in early 1921, five months before the onset of the Rif War. Invited by the Professor of Gynaecology and Obstetrics and Dean of Madrid’s Faculty of Medicine Sebastián Recasens, Chutró stayed in the Spanish capital on 13–18 February. He performed some operations at the Madrid-Carabanchel Hospital and gave four scientific lectures with great success. Two of them must have sparked Bastos’ interest, the one at the Faculty of Medicine (Figure 7) on ‘fractures of the thigh’ and the one at the Army Medical Academy on ‘sequelae following bone trauma’.^108^ In the first lecture, Chutró advocated for his long-established continuous traction procedure for open fractures of the femur and tibia with only some minor modifications.^109^ In the second, he dealt with pseudarthrosis following fractures of the humerus. After describing the various available procedures to prevent this problem (ligatures, metal plates, bone grafts), he nevertheless held, as he had done in 1918 at the *Société de Chirurgie*, to the belief that, compared to the femur and even to the forearm bones, the fractured humerus consolidated ‘easily […] with almost nothing, with a ligature and a plaster cast’,^110^ provided that this cast was not applied through the device ‘used by the [North] Americans in fractures of the arm and known as “airplane”’, for this ‘causes a vicious abduction of the traumatised arm, with deviation of the scapula outwards, exceeding the costal line’.^111^

We believe this lecture by Chutró, then at the zenith of his career, personality, and fame, convinced Bastos of the value of the occlusive approach in compound humerus fractures and helped him to overcome his apprehension towards it when the Rif War broke out six months later. Beyond the personal impact, Chutró’s influence on Bastos must be framed within the general adoption by the Army Medical Service during the Rif War of many organisational and technical elements that the belligerent armies had developed during the First World War. Surgical teams^112^ and blood transfusion^113^ were some of the most important, but also, more specifically for Bastos, base hospitals and fracture centres.^114^ The adoption of the closed approach to fractures was also due to the characteristics of warfare in Morocco. Even in the hardest period of combats between September 1925 and May 1926, many of the arm wounds continued to be linear because of the predominance of Mauser rifles over artillery and machine guns among the Riffians. Finally, the application of this procedure was favoured by the faster evacuation of the wounded, thanks to improvements in immobilisation, in the delivery of emergency surgery at the front (after the creation of first aid posts and mobile field hospitals), and in the use of ambulance cars, hospital trains and ships, and medical aircraft.

## Neglected transatlantic circulations

Chutró was not just influential for Bastos’ practice of the closed method, but also for the publication of his first results. Two years on in the Rif War, he had not yet written any paper, probably due to wartime overwork, the need for prolonged follow-up of cases, the direction of the new rehabilitation service, etc. The expected attendance of Chutró at the 2nd National Congress of Medical Sciences scheduled for October 1924 in Seville broke Bastos’ impasse. As early as August 1923, Recasens, the head of the organising committee (he was born in Seville), was charged by King Alfonso XIII with crossing the Atlantic to ensure that a large representation of South American doctors attended the event, thereby reflecting the importance that the monarch attached to ‘everything related to the intellectual exchange between Spain and Latin America’.^115^ The coming to power of Primo de Rivera after his *coup d’etat* in September only reaffirmed this plan, which was so successful that the congress, albeit national (Spanish), would be alternatively called ‘1^st^ Ibero-American Congress of Medical Sciences’,^116^ ‘Congress of Hispano-American and Luso-Brazilian Medical Sciences’,^117^ and even ‘Hispano-Luso-American Congress’.^118^ Bastos, whose institutional rapprochement with Recasens had resulted in his appointment as a member of the organising committee,^119^ must have known very early that Chutró and other prominent Argentinian surgeons had accepted the invitation.^120^ Since Chutró’s visit in 1921, he had grown used to meeting visitors from the River Plate country. Avelino Gutiérrez, for example, a Spanish emigree who worked at the Spanish Hospital in Buenos Aires and was president of the *Asociación Cultural Española de Argentina* [Spanish Cultural Association in Argentina],^121^ visited Madrid in 1923, giving lectures on the topographical and operative anatomy of the neck that Bastos attended.^122^ In January 1924, Enrique Finochietto, as renowned as Chutró, for ‘his studies on the treatment of fractures during the Great European War’, was in the Spanish capital to close a long stay in Europe that took him to Paris and other cities. He gave a lecture at the Royal Academy of Medicine on hydatid cysts of the lung^123^ and operated in several surgical centres,^124^ among which the Madrid-Carabanchel Hospital, where Bastos welcomed him at his service and invited him to perform ‘the most difficult operations […] but he modestly chose those of simpler execution’.^125^

The expected attendance of Chutró, Avelino and Alberto Gutiérrez (father and son), and other figures of Argentinian surgery to the organised-from-Madrid-held-in-Seville congress must have moved Bastos to publish the results of his work with the wounded in Morocco. The year 1924 became one of the most prolific in his career, which was never characterised by an excess of publications. He released two papers on the treatment of fractures in the lower and upper limbs and another one about orthopaedic surgery, all three in Spanish journals.^126^ The second in chronological order was his decisive *Tribuna* article of October 1924. There, Bastos quoted Chutró explicitly when he explained that defective immobilisation was behind

the lack of consolidation and pseudarthrosis. The humerus, like the tibia in its anterior part, has a very poor periosteum, probably due to the large surface area of free bone, i.e. without muscular insertions. There also seems to be a vascular arrangement in the arm, well studied by Chutró and [Erich] Lexer, which favours local necrosis of the fracture surfaces.^127^

This article saw the light coinciding exactly with Chutró’s stay in Spain. The Argentinian surgeon disembarked in Cadiz at the end of September and, after a tour of Andalusia, travelled to Madrid to give some lectures before moving on to Seville. Chutró lectured in Madrid on 1 and 2 October on the clinic and treatment of acute osteomyelitis.^128^ He also operated at Madrid-Carabanchel, where, according to the press chronicles, he ‘poured out his great mastery as a surgeon […] generously […] on the “little Spanish soldiers”, as he called them, wounded in Africa, where our army doctors, eager for these teachings, studied and admired him’.^129^ Bastos, always open and supportive to the exchanges between military and civilian medicine, attended as a ‘captivated listener’^130^ to these lectures, which dealt with a subject of direct interest to him. Both surgeons personally interacted with each other, first in Madrid, then in Seville (where, surprisingly, Chutró did not present any paper) and a few weeks later again in Madrid, where Chutró gave three new lectures on 20–22 October.^131^ It seems plausible to think that Bastos took the chance to hand Chutró a copy of his paper.

It also seems, however, that personal contact did not have any effect as far as the closed method was concerned. In fact, Chutró devoted his gradually less frequent publications in the following years to topics ever more distanced from those which had given him international fame. Bastos, meanwhile, spent years focused on orthopedic surgery and rehabilitation before taking fractures and the closed method back as a main subject of research and publication. The two surgeons may have gotten along not so well with each other. In his *Memorias*, Bastos pictured the Argentinian as a ‘big-headed, dour man’, despite recognising him as a great teacher and a ‘pioneer’.^132^ He also downplayed Chutró’s impact in Spain by limiting his influence to practical surgical matters such as the adequate illumination of the operating room and the use of blue-, grey- or green-coloured linen instead of white, leaving unexplained in his book ‘the many things no less useful and of no lesser evidence that Chutró placed before our eyes’.^133^ This contrasts greatly with his warm memories of other Argentinian surgeons who visited Madrid^134^ before attending the Seville congress, such as Ángel Alsina, Alejandro Ceballos, the aforementioned Alberto Gutiérrez,^135^ or Enrique Finochietto’s brother, Ricardo, who ‘spent some time at my clinic as if he were a regular collaborator’.^136^ In any case, Chutró and the other Argentinian doctors turned Madrid into a crossroads in the circulation of European and American surgical practices during the 1920s, of lesser importance than Paris, London, or Berlin, but still relevant, especially because of connections with Latin America and the impact of the Rif War. Bastos had a significant role in this process thanks not just to his work with Moroccan wounded and his reception of Argentinian visitors, but also to the invitations he personally issued to figures of traumatology and rehabilitation that accepted to visit him in Madrid, such as the Austrian Hans Spitzy, the Germans Friedrich Ernst Krukenberg and Otfried Proebster, ‘Reynaldo dos Santos and [Francisco] Gentil, from Portugal, […] Araújo from Brazil, Doctors Beckers, Mayer and Van der Velde, from Belgium, [and Dr.] Rocher, from France’.^137^

Chutró would return one last time to Spain in 1929. Invited again by Recasens, he stayed for over a month operating and giving lectures.^138^ In a clinical session at the Madrid-Carabanchel Hospital, he dealt once more with the pseudarthrosis of the humerus, analysing its causes and explaining its treatment with bone grafts.^139^ Bastos, who attended the session as on previous visits, had become by then an established figure. Apart from his rehabilitation service at the military hospital, he directed another one in Madrid’s Provincial Hospital of San Carlos and had become a lecturer at the university. Thanks to this, he built a team of civilian and military disciples in orthopaedics and traumatology. Bastos’ institutional insertion had owed much to his mentor López-Durán, whom he also replaced at the *Instituto Rubio* after his decease in 1930. During the period of the Second Republic (1931–36), Bastos travelled to Argentina to participate in the 2nd National Congress of Medicine held in Rosario in 1934. In Buenos Aires he operated and gave lectures at the Academy of Medical Sciences, the Spanish Hospital, the Military Hospital, and the Faculty of Medicine. Although he must have met colleagues who had visited him in Madrid in previous years, he did not mention any of them in his *Memorias.* ^140^ Neither did he mention Chutró, who died not long afterwards, in 1937.^141^

**Figure 8.**
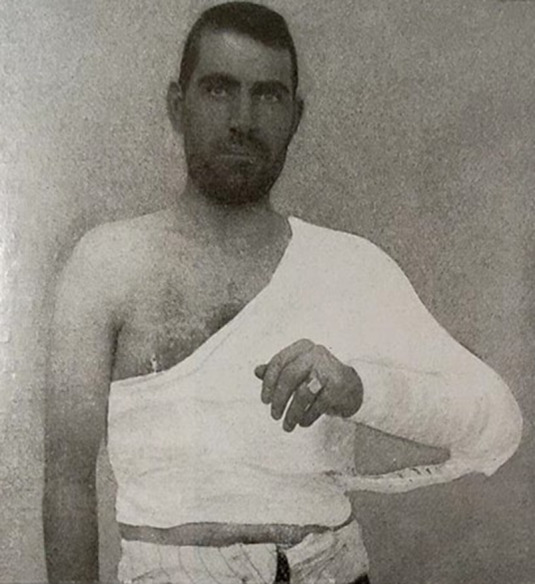
Closed method treatment of humerus fracture in a soldier wounded at the 1934 Asturias Revolution. Source: Bastos, 1936, 63. Francisco Javier Martínez’s private library.

No sooner had Bastos returned from that trip, a revolutionary strike broke out in Spain, with bloody clashes in the northern region of Asturias between miners and the army. Bastos practised the closed method in wounded soldiers, this time not only in fractures of the humerus (Figure 8) but also of the forearm and even of the femur ‘in well-defined cases of uncomplicated war fractures with linear trajectory’.^142^ These (not quantified) cases, with others from the Rif War, were included in his already mentioned treatise *Algunos aspectos clínicos de las heridas por arma de fuego* published a few months before the Civil War broke out. As said before, Bastos acknowledged there that he followed ‘Orr’s prescriptions’,^143^ but the absence of bibliographic references in the book makes it impossible to know to which publications of the American surgeon he was referring. Neither is it possible to know, for the same reason, in what esteem Bastos held his own pioneering article of 1924 or Chutró’s work. The book widely circulated before and during the Spanish Civil War, serving as a reference for surgeons on both sides. However, when the war was over, Bastos fell into oblivion. Trueta, D’Harcourt, and other Spanish surgeons did not reclaim his pioneering work. He was removed from the Army Medical Service by Francoist authorities and became a domestic exile, earning his life with the translation of medical treatises and private practice. He never again published on the closed method until his death in 1973.

## Conclusion

Our study on Manuel Bastos has tried to ‘open’ the established historical narrative of the closed method by presenting him as a major actor and also by applying novel analytic frames. Instead of taking it as a single, univocally traceable procedure, we believe the closed method was a multidimensional development, with parallel versions emerging in different locations and transforming themselves and influencing each other as they circulated globally and were put to practice in various wars. As David Edgerton or Ilana Löwy have argued, innovations have an essentially collective character and the depiction of how some procedures come to be accepted and their authorship attributed in different historical contexts is also more important than authorship claims.^144^ For the closed method, while the global crossroads of First World War Paris seems to have been the common source of all versions, the subsequent fate of each one depended on a multitude of factors, from the personal circumstances and scientific abilities of individual surgeons, to the degree of development of medicine and healthcare in their respective countries, to the greater or lesser impact of dissemination channels (journals, congresses, media) and the outcome of revolutions and wars in which they got involved. In this sense, the reconstruction of Manuel Bastos’ early trajectory and practice of the period 1909–1924 has put forward one of those lesser-known versions of the closed method with its own distinctive roots and features. Firstly, the prominence of military medicine in Spain and the fluid exchanges with civilian medicine (including non-governmental reformist institutions) that underlay the collaboration between Bastos and his mentor, Adolfo López-Durán. Secondly, the importance of the Rif War for Spain, a sort of belated First World War or anticipated Spanish Civil War in terms of the scale of military operations, the incorporation and development of medical and sanitary innovations, and the high number of dead, wounded, and sick. Finally, the neglected role of global circulations, embodied by Pedro Chutró’s trans-Atlantic mobilities between Argentina, France, the United States, and Spain and his impact on Bastos within a revival of Hispano-Americanism and in particular of Spanish-Argentinian exchanges.

## Footnotes

1

Hiram Winnett Orr , ‘The Treatment of Fractures’, Journal of Orthopedic Surgery, 3, 1, (1921a), 23–27 ; ‘Early and Complete Immobilization as a Factor in the Preservation of Joint Function in the Treatment of Fractures’, *American Journal of Surgery*, 35, 146 (1921b), 67–75; *An orthopedic surgeon’s story of the Great War* (Lincoln: [s.n.], 1921c); ‘The Fundamental Principles of Orthopedic, Reconstruction and Industrial Surgery. The Real Lessons of the World War’, *Journal of the American Medical Association* (henceforth, JAMA), 79, 4 (1922), 255–257; ‘A New Method of Treatment for Chronic Infections Involving Bone’, *The Nebraska State Medical Journal*, VIII, 2 (1923), 50–52; with J.E.M. Thomson, ‘Maintaining Length and Position in the Treatment of Compound Fractures’, *JAMA*, 83, 18 (1924), 1399–1403.

2

Patrocinio Moratinos et al, ‘El “método español” de tratamiento de las heridas de guerra creado y desarrollado en la Escuela de Cirugía del Hospital Militar de Madrid-Carabanchel entre 1921-1936 por el insigne médico militar D. Manuel Bastos Ansart. Repercusión internacional’. Revue internationale des services de santé des forces armées, 74, 1 (2001), 49–67 .

3

Josep Trueta , ‘“Closed treatment” of War Fractures’, The Lancet, 233, 6043 (1939a), 1452–5; ‘The Closed Plaster Method’, *The Lancet*, 236, 6098 (1940a), 53.

4

Josep Trueta , ‘Treatment of War Wounds and Fractures’, British Medical Journal, 1, 4245, (1942), 616–1720784237 10.1136/bmj.1.4245.616PMC2160834.

5

Josep Trueta , Treatment of War Wounds and Fractures: With Special Reference to the Closed Method as Used in the War in Spain (London: Hamish Hamilton, 1939b).

6

Josep Trueta , Treatment of War Wounds and Fractures: With Special Reference to the Closed Method as Used in the War in Spain (New York: Paul B. Hoeber, 1940b).

7 Trueta, *op. cit.* (note 4).

8

L.W. Plewes , ‘Remarks on the Orr-Trueta Treatment’, Canadian Medical Association Journal, 44, 2 (1941), 193–420322012 PMC1826869; C. Giannou , M. Baldan , A. Molde , War Surgery. Working with Limited Resources in Armed Conflict and Other Situations of Violence, vol. 2 (Geneva: ICRC, 2013).

9

Antonina Rodrigo , Josep Trueta. Héroe anónimo de dos guerras (Barcelona, Plaza & Janés, 1977); Gabriel Jackson , ‘Josep Trueta and the Significance of Catalan Nationality’, The Centennial Review, 23, 3 (1979), 309–28; Felip Cid , La contribució cientifica catalana a la medicina i cirurgia de guerra (Barcelona: Fundación Uriach 1838, 1996); Oriol Cassassas , Josep Trueta i Raspall, el símbol. Semblança biogràfica de Josep Trueta i Raspall (Barcelona: Institut d’Estudis Catalans, 2004); Elena Guardiola , Eladi Baños , ‘El mètode Trueta’, *Quaderns de la Fundació Dr*
. Antoni Esteve, 1, (2011) 91–5.

10 For example, in the international workshop *War Surgery: Josep Trueta and Experimental Medicine (1921–1945)* held in Barcelona in 2019, there were presentations on the practice of occlusive treatments in France, Germany, Austria, the USSR and Spain (including Franco’s side in the Civil War) during the period 1921–45. https://www.residencia-investigadors.es/ca/activitats/cirurgia-de-guerra-josep-trueta-i-la medicina.html (accessed 7 May 2025).

11

Roger Cooter , Mark Harrison , Steven Sturdy , War, Medicine and Modernity (Sutton: Stroud, 1998); Leo van Bergen , ‘The Value of War for Medicine: Questions and Considerations Concerning an Often-endorsed Proposition’, Medicine, Conflict, and Survival, 23, 3 (2007), 189–9717822062 10.1080/13623690701417337.

12

Bruno Latour , Pasteur, guerre et paix des microbes (Paris: La Découverte, 2001); Kapil Raj , Relocating Modern Science: Circulation and the Construction of Knowledge in South Asia and Europe, 1650–1900 (New York: Palgrave Macmillan, 2007).

13

Joaquín Salarich , ‘Nota biográfica del doctor Don Manuel Bastos Ansart’, Anales de Medicina y Cirugía, LIII, 232 (1973), 187–94; José Manuel Gómez López , ‘Notas para la biografía personal y científica de Manuel Bastos Ansart (1887-1973)’, Gimbernat, 46 (2006), 183–96; Ángel Beneito Lloris , ‘El Dr. Manuel Bastos, profesor y militar represaliado’, in Josep Lluís Barona (ed.) El exilio científico republicano (Valencia: Universidad de Valencia, 2010), 315–32; *El hospital Sueco Noruego de Alcoi durante la Guerra Civil Española* (Alcoi: Visual Producciones, 2004).

14

Juan Moral Torres , ‘El “método español” en el tratamiento de las heridas de guerra’, in Los médicos y la medicina en la Guerra Civil Española (Madrid: Laboratorios Beecham, 1986), 157–66; Patrocinio Moratinos et al, ‘Antecedentes en la escuela clásica española de cirugía militar conservadora del “método español” de tratamiento de las fracturas abiertas, desarrollado por el Dr. Manuel Bastos Ansart en el Hospital Militar de Madrid-Carabanchel, entre 1921 y 1936’, Medicina Militar, 54, 3 (1998), 183–8; Moratinos, *op. cit.* (note 2); Alejandro Belaústegui , Sanitarios militares en la Guerra de África, 1909-1927. La lucha contra el olvido VI (Madrid: Ministerio de Defensa, 2011).

15 Alfons Fernández Sabaté, *Nuestros fundadores y maestros en 1935 y 1945* (Madrid: SECOT, 2003); Cassassas, *op. cit.* (note 9), 9.

16 Moratinos Palomero, *op. cit.* (note 2), 16.

17 Cid, *op. cit.* (note 9), 110.

18

Guillermo Carroquino , ‘Manuel Bastos Ansart, un pionero de la traumatología militar y de la cirugía ortopédica española’, in Sanidad Militar. 500 años al servicio de España (Madrid, Ministerio de Defensa, 2015), 210 .

19

Francisco Jimeno Vidal , ‘Algunas anotaciones para la pequeña historia del vendaje enyesado’, in Libro homenaje al profesor doctor D. Manuel Bastos Ansart (Madrid: SECOT, 1973), 180–9, p. 189.

20 Moral Torres, *op. cit.* (note 15), 161.

21 Cid, *op. cit.* (note 9), 119.

22

Manuel Bastos , De las guerras coloniales a la Guerra Civil. Memorias de un cirujano (Barcelona: Ariel, 1969), 171 .

23

Georges Gusdorf , ‘Conditions and limits of autobiography’, in James Olney (ed.) Autobiography (New Haven: Princeton University Press, 1980), 28–48 ; Dorinda Outram , ‘Life-paths: autobiography, science and the French Revolution’, in Michael Shortland , Richard R. Yeo (eds.) Telling Lives in Science. Essays on Scientific Biography (Cambridge: Cambridge University Press, 1996), 85–102 ; Alexia Sanz , ‘El método biográfico en investigación social: potencialidades y limitaciones de las fuentes orales y los documentos personales’, Asclepio, LVII, 1 (2005), 99–115 .

24

Francisco Javier Martínez , Matheus Duarte da Silva , ‘Disease landscapes beyond the Spanish flu pandemic: temporal patterns, re-centered narratives (1889-1970s)’, Dynamis, 45, 1 (2025), 11–25 .

25

Roger Cooter , ‘The meaning of fractures: orthopaedics and the reform of British hospitals in the inter-war period’, Medical History, 31 (1987), 306–323306221 10.1017/s0025727300046883PMC1139743.

26

Francisco Javier Martínez , ‘Public health and empire in Isabelline Spain (1833-1868): the case of military health’, Història, ciência, saúde. Manguinhos, 13, 2 (2006), 439–75.

27

María Rosa de Madariaga , ‘La guerra de Melilla o del Barranco del Lobo, 1909’, in Eloy Martín-Corrales (ed.) Semana Trágica. Entre las barricadas de Barcelona y el Barranco del Lobo (Barcelona: Bellaterra, 2011), 112 .

28

Javier Martínez , Alejandra de Leiva , ‘Lazos de sangre: la Guerra del Rif y la transfusión sanguínea en España’, in Javier Martínez (ed.) Horrores cercanos. Guerra e innovación humanitaria en la España contemporánea, 1860-1939 (Madrid: Libros de la Catarata, 2024), 79 .

29 Bastos, *op. cit.* (note 23), 74.

30

Sebastián Lazo , ‘La medicina militar española en el Rif’, Los progresos de la clínica, 2, 1 (1913), 228 .

31

Clinton T. Dent , William Thomson , ‘The War in South Africa’, The British Medical Journal, 1, 2051 (1900), 968–74.

32

Rafael Ramírez-Rivas , ‘Notas clínicas. Heridas graves de antebrazo derecho en cirugía de guerra’, Los Progresos de la Clínica, 4, 2 (1915), 120 .

33
*Ibid.*, 121.

34 Bastos, *op. cit.* (note 23), 93.

35

Josep Maria Massons , Historia de la Sanidad Militar española (Barcelona, Ed. Pomares-Corredor, 1994).

36

Juan Luis Carrillo-Martos , Medicina y sociedad en la España de la segunda mitad del siglo XIX: una aproximación a la obra de Federico Rubio y Galí, (1827-1902) (El Puerto de Santa María: Ayuntamiento del Puerto de Santa María, 2003).

37

Antonio Jiménez Landi , La Institución Libre de Enseñanza y su ambiente (Madrid, Editorial Complutense, 1996).

38 ‘Adolfo López-Durán’. Médicos históricos españoles, Universidad Complutense de Madrid. https://medicoshistoricos.ucm.es/s/medes/item/837203 (accessed on May 7, 2025).

39 For example, his treatise *Notas clínicas de cirugía ortopédica* (1911, 1913) was reviewed in the *American Journal of Orthopedic Surgery*, *The Lancet* and *Zeitschrift für orthopädische Chirurgie.*

40

Adolfo López-Durán , Manuel Bastos , ‘Tratamiento de las fracturas de la extremidad superior del húmero’, Los Progresos de la Clínica, 3, 4 (1914), 288–99.

41

Adolfo López-Durán , Manuel Bastos , La práctica del tratamiento de las fracturas de los miembros (Madrid: Librería Gutenberg de José Ruiz, 1915), 12–18 .

42
*Ibid.*, 18–21.

43

Leo Van Bergen , Before my Helpless Sight. Suffering, Dying and Military Medicine on the Western Front, 1914-1918 (London: Ashgate, 2009), 1–30 .

44

Summer Koch , ‘Biographical sketch’, in A Catalogue of the H. Winnett Orr Historical Collection and Other Rare Books in the Library of the American College of Surgeons (Chicago: American College of Surgeons, 1960), x.

45 Idem.

46 Orr, *op. cit.* (note 1), 1921a, 36.

47 Idem.

48
*Ibid.*, 46. Italics are of the authors.

49

Leo van Bergen , ‘The Value of War for Medicine’, Medicine, Conflict and Survival, 23, 3 (2007), 194 10.1080/1362369070141733717822062.

50 Orr, *op. cit.* (note 1), 1923, 51.

51 Idem.

52 Idem. Italics are of the authors.

53
*Ibid.*, 52.

54 Orr, Thomson, *op. cit.* (note 1), 1401.

55

Hiram Winnet Orr , ‘Treatment of Osteomyelitis and Other Infected Wounds by Drainage and Rest’, Surgery, Gynecology & Obstetrics, 45, 466 (1927), 446–64; ‘The Treatment of Infected Wounds without Sutures, Drainage Tubes or Antiseptic Dressings’, *The Journal of Bone and Joint Surgery*, 10, 3 (1928), 605–11; *Osteomyelitis and Compound Fractures* (St. Louis: C. V. Mosby Company, 1929).

56 José Luis de Mesa, Carlos Lázaro-Ávila, Roberto Muñoz-Bolaños, Jesús Núñez, Las campañas de Marruecos, 1909-1927 (Madrid: Almena, 2001); Roberto Muñoz Bolaños , ‘El Imperio Español Contraataca’, in Daniel Macías (ed.) A cien años de Annual. La guerra de Marruecos (Madrid: Desperta Ferro, 2021), 229–79.

57 Martinez, de Leiva, *op. cit.* (note 27), 78–82.

58

Manuel Bastos , ‘La práctica del tratamiento de las fracturas abiertas del muslo’, Los Progresos de la Clínica, 2 (1924), 131 .

59 Bastos, *op. cit.* (note 23), 170.

60
*Ibid.*, 171.

61

Manuel Bastos , La práctica del tratamiento de las fracturas de húmero abiertas o cerradas. (Trabajo publicado en Tribuna Médica Española, octubre 1924) (Madrid: Artes Gráficas Plus Ultra, 1925).

62 Bastos, *op. cit.* (note 58), 3.

63
*Ibid.*, 12.

64 Idem.

65 Idem.

66
*Ibid.*, 14.

67
*Ibid.*, 26.

68
*Ibid.*, 22–3.

69
*Ibid.*, 24.

70
*Ibid.*, 19.

71 Bastos, *op. cit.* (note 23), 170.

72 López-Durán, Bastos, *op. cit.* (note 37), 20.

73

Manuel Bastos Ansart . Algunos aspectos clínicos de las heridas por arma de fuego (Barcelona: Labor, 1936), 24 .

74 Joaquín D’Harcourt, A. Folch i Pi, A. Oriol i Anguera, ‘La cure occlusive en chirurgie de guerre’, Revue de Chirurgie, 58, LXXVII (1939), 645. D’Harcourt forgot to mention the article that Bastos had published in *Tribuna Médica Española.*

75 Josep Trueta, *Fragments d’una vida* (Barcelona: Edicions 62, 1980), 95.

76 Orr, *op. cit.* (note 1), 1922, 256.

77 Bastos, *op. cit.* (note 23), 184.

78

José Ignacio Allevi , Stefan Rinker (eds.), Saberes globales y expertos locales en América Latina en el siglo XX (Freiburg: Herder, 2024).

79

Álvaro Girón , Oliver Hochadel , Gustavo Vallejo (eds.), Saberes transatlánticos. Barcelona y Buenos Aires. Conexiones, confluencias y comparaciones, 1850-1940 (Madrid: Doce Calles, 2017).

80

Susana Sueiro Seoane , ‘Retórica y realidades del “Hispanoamericanismo” en la Dictadura de Primo de Rivera’, Mélanges de la Casa de Velázquez, 28, 3(1992), 143–59; Eduardo González Calleja , La España de Primo de Rivera. La modernización autoritaria, 1923-1930 (Madrid: Alianza, 2005), 125–7.

81

Juan Pablo Fusi , Antonio López Vega (eds.), Diálogos atlánticos. Cultura y ciencia en España y América en el siglo XX (Madrid: Galaxia Gutenberg, 2021).

82 Of 1,176 registrations to the congress (most of them individual physicians, but also institutions, laboratories, etc.), over 160 (13%) were from Argentina, practically all from Buenos Aires. *II Congreso Nacional de Ciencias Médicas y Exposición Aneja* (Madrid-Barcelona, Los Tiroleses, 1924), 83–139.

83 ‘II Congreso Nacional de Ciencias Médicas’, *Revista de Sanidad Militar*, XIV, 11 (1924), 297.

84 Mario Rubén Sanguina, ‘Aporte sanitario de la comunidad argentina a Francia durante la Primera Guerra Mundial: 1914-1918’ (Société Archéologique et Historique de Clermont en Beauvaisis, 2019) https://efran.cancilleria.gob.ar/userfiles/2018/medicos_voluntarios_argentinos_14-18_en_castellano1.pdf (accessed on May 25, 2025)

85

Pierre Fredet , ‘Notice nécrologique sur M. Pedro Chutró (1880-1937)’, Bulletin de l’Académie Nationale de Médecine, 120, 1 (1938), 26 .

86 Alfredo Buzzi S.f., ‘Pedro Chutró. Figura de la medicina argentina’, *Alma. Cultura & Medicina* http://www.almarevista.com/revista/sin-categoria/eduardo-braun-menendez/ (accessed on May 28, 2021).

87 ‘Séance du 20 Décembre 1916’, *Bulletins et mémoires de la Société de Chirurgie de Paris*, XLIII, 1 (1917), xxxi.

88

Anne Rasmussen , ‘Science and Technology’, in John Horne (ed.) A Companion to World War I (Malden-Massachusetts: Wiley-Blackwell, 2010), 307–22.

89 Martínez, de Leiva, *op. cit.* (note 27), 75–6.

90

United States War Department. General orders (Washington: Government Printing Office, 1920), 6 .

91 Orr, *op. cit.* (note 1), 1921, 41.

92

Alfred Pearce Gould et al. ‘The War. Carrel-Dakin Treatment of Wounds’, British Medical Journal, 2 2966 (1917), 597 20768800 .

93 Idem.

94 Idem.

95

Pedro Chutró , ‘Pseudoarthrose de l’humerus’, Bulletin et Mémoires de la Société de Chirurgie de Paris, XLIV, 3 (1918), 147–8.

96

F. Chauvin , L.P. Fischer , J.J. Ferrandis , ‘L’évolution de la chirurgie des plaies de guerre des membres en 1914-1918’, Histoire des Sciences Médicales, 36, 2 (2002), 170 12387254.

97

William D. Mayo , ‘Medical conditions in South America’, Bulletin of the Pan-American Union, 52 (1921), 55 .

98 Fredet, *op. cit.* (note 82), 27.

99

Steven J. Peitzman , ‘”Thoroughly practical”: America’s Polyclinic Medical Schools’, Bulletin of the History of Medicine, 54, 2 (1980), 168 6994847.

100

Pedro Chutró , ‘Bone Fistulas after War Wounds’, JAMA, 73, 10 (1919), 751–2.

101

E. I. Salisbury , ‘The Late Treatment of War Osteomyelitis’, United States Naval Medical Bulletin, 14, 1 (1920), 255–6.

102 ‘Proceedings of the Atlantic City Session’, *JAMA*, 72, 26 (1919), 1915.

103 Fredet, *op. cit.* (note 82), 27.

104 Enrique Finochietto was another renowned Argentine surgeon who during the First World War took over the direction of the Argentine Hospital in Paris. *Caras y Caretas*, August 9, 1919. José de Sard , ‘Conferencia del Dr. de Sard en la Facultad de Medicina de Madrid con motivo del I Congreso Nacional de Medicina’, Revista de Sanidad Militar, IX, 11 (1919), 321 .

105

George W. Hawley , ‘A Combination Finochietto and Steinmann Pin Traction Stirrup’, JAMA, 70, 1 (1918), 23 .

106 Bastos, *op. cit.* (note 23), 125.

107 In his *Memorias*, Bastos recalled a private visit to Paris in the spring of 1920 in which ‘everywhere we met Spaniards or people who spoke Spanish, especially Argentinians. The latter were the object of a veritable cult in Paris, and all Spanish speakers participated in it’ Bastos, *op. cit.* (note 23), 129. Perhaps he also heard praise of Chutró’s wartime work, only a few months before the Argentinian returned to the French capital.

108 Chutró gave a second lecture at the Faculty of Medicine on ‘The role of the abdominal wall in inguinal hernias and eventrations’ and another at the Royal Academy of Medicine on ‘Relations between ptosis of the kidney and lesions of the right colon’. *El Sol*, February 17, 1921; *La Acción*, February 18, 1921.

109

Jesús Riosalido , ‘El doctor Chutró’, Revista Iberoamericana de Ciencias Médicas, VII, 71 (1921), 1067–70.

110
*La Correspondencia Militar*, February 18, 1921.

111 Ibidem.

112

Luis Alfonso Arcarazo , ‘El Cuerpo de Sanidad Militar en las campañas de Marruecos’, Revista de Historia Militar, 93 (2003), 185–243 .

113 Martínez, de Leiva, *op. cit.* (note 27).

114 Manuel Bastos, *op. cit.* (note 55), 132.

115
*El Financiero*, September 28, 1923.

116 Joaquín Herrera Carranza, ‘La Federación Sanitaria de Andalucía (1916-1929). Un proyecto hispalense’, *Archivo Hispalense*, 279-281 (2009), 78.

117 Eduardo Navarro Salvador. ‘La raza hispana y el Congreso de Sevilla’. *El Carbayón*, October 31, 1924. https://www.filosofia.org/hem/dep/car/9241031.htm [accessed June 25, 2025].

118

José de Lignières , ‘Informe de la delegación al I Congreso Hispanolusoamericano de Ciencias Médicas en Sevilla (octubre de 1924)’, Revista de la Facultad de Agronomía y Veterinaria, 5, 2 (1926), 205–9.

119 Bastos, *op. cit.* (note 23), 168.

120
*El Financiero*, September 28, 1923.

121 This association promoted scientific and cultural links between both countries. Between 1912 and 1920, Avelino, his brother Ángel and his son Alberto had funded and organised homages to Spanish figures such as Marcelino Menéndez Pelayo, Santiago Ramón y Cajal, Ramón Turró, and Joaquín Costa, and granted aids for the training of young Spanish students abroad. *La Industria Nacional*, February 28, 1924.

122
*El Financiero*, September 18, 1923; Bastos, *op. cit.* (note 23), 185.

123
*La Acción*, December 31, 1923.

124
*La Acción*, December 25, 1923.

125 Bastos became a member of the *Real Academia de Medicina* in January 1924 at the same session in which Finochietto was elected as foreign correspondent. *El monitor sanitario*, 1924, 141.

126 Bastos, *op. cit.* (note 56); *op. cit.* (note 53); ‘Los resultados de la cirugía de rehabilitación en nuestros inválidos de guerra’, *Anales de la Real Academia Nacional de Medicina*, XLIV (1924), 602–10. In addition, at the Seville congress, he made two presentations, one about ‘Surgery of the nerve trunks’, in the section ‘General Surgery’ chaired by his former teacher at Zaragoza Ricardo Lozano Monzón, and in which Enrique Finochietto also presented several papers; and the second on ‘Cineplasties’ in the section ‘Surgery of accidents. Orthopaedics. Mecanoteraphy’ chaired by his mentor López-Durán. *Op. cit.* (note 79), 35, 39.

127 Bastos, *op. cit.* (note 58), 3.

128
*Heraldo de Madrid*, October 2, 1924.

129
*El Imparcial*, October 9, 1924.

130 Bastos, op. cit. (note 23), 185.

131
*El Imparcial*, October 9, 1924. The topics of the lectures were: ‘The first hours of acute appendicitis’ (20th October), ‘Joint deformations in tuberculous osteoarthritis’ (21st) and ‘Examination of tuberculosis of the knee or hip’ (22nd).

132
*Ibid.*, 185.

133 Bastos, *op. cit.* (note 23), 184-185.

134
*La Voz*, October 9, 1924; Bastos, *op. cit.* (note 22), 185.

135 Gutiérrez, thanks to his profound knowledge of Spanish military medicine, would play a decisive role many years later in the recognition of the priority of the contributions of the Spanish army doctor Fidel Pagés, a graduate in Zaragoza like Bastos, in the discovery of epidural anaesthesia over the Italian doctor Achille Dogliotti. María Senovilla , ‘Cien años sin dolor’, Revista Española de Defensa, 12 (2020), 54–7.

136 Bastos, *op. cit.* (note 23), 185.

137
*Ibid.*, 186.

138
*La Libertad*, February 3, 1929.

139

Mariano Madruga . ‘El Dr. Chutró en el Hospital Militar de Carabanchel’. Revista de Sanidad Militar, XIX, 3 (1929), 80–1.

140 Bastos, *op. cit.* (note 23), 197–209.

141 Fredet, *op. cit.* (note 82).

142 Bastos, *op. cit.* (note 70), 25, 39, 59, 63.

143
*Ibíd.*, 24.

144

Ilana Löwy (ed) Medicine and Change: Historical and Sociological Studies on Medical Innovation (Montrouge: John Libbey Eurotext-Inserm, 1993); David Edgerton . ‘Innovation, Technology or History. What Is the Historiography of Technology About?’. Technology and Culture, 51, 3 (2010), 680–97.

## Long descriptions

### Figure 2. Long description

A geographical map of the Strait of Gibraltar and Northern Africa.

* North: The southern coast of Spain is visible with labels for Cádiz, Algeciras, Málaga, and Gibraltar U.K. The Mediterranean Sea lies to the Northeast.

* Center: The territory of Spanish Morocco is highlighted in orange, stretching along the northern coast. Key cities labeled within this orange zone include Ceuta, Tétouan marked with a star, Chechaouene, Al Hoceima, Melilla, and Nador. The word Rif is printed across the eastern portion of this zone.

* Northwest: A small red-shaded area on the coast is labeled Tangier International Zone, with Tangier marked by a star.

* South: The region below the orange zone is shaded green, representing the rest of Morocco. Cities labeled in this green area include Ouezzane, Taounate, Taza, Oujda to the East, and the coastal cities of Kenitra, Salé, Rabat, and Casablanca to the Southwest. Inland cities Fès and Meknès are also marked.

Navigate back to Figure 2..

